# Development and Validation of Surveys to Estimate Food Additive Intake

**DOI:** 10.3390/nu12030812

**Published:** 2020-03-19

**Authors:** Gina L. Trakman, Winnie Lin, Amy L. Wilson-O’Brien, Annalise Stanley, Amy L. Hamilton, Whitney Tang, Leo Or, Jessica Ching, Mark Morrison, Jun Yu, Siew C. Ng, Michael A. Kamm

**Affiliations:** 1 Department of Gastroenterology, St Vincent’s Hospital, Melbourne 3065, Australia; G.Trakman@latrobe.edu.au (G.L.T.); amy.wilson-obrien@svha.org.au (A.L.W.-O.); annalise.stanley@svha.org.au (A.S.); Amy.HAMILTON@svha.org.au (A.L.H.); 2 Department of Medicine, The University of Melbourne, Melbourne 3052, Australia; 3 Department of Dietetics, Nutrition and Sport, La Trobe University, Melbourne 3086, Australia; 4 Department of Medicine and Therapeutics, The Chinese University of Hong Kong, Shatin, NT, Hong Kong SAR, China; winnielin@cuhk.edu.hk (W.L.); whitneytang@cuhk.edu.hk (W.T.); leoor@cuhk.edu.hk (L.O.); jessicaching@cuhk.edu.hk (J.C.); junyu@cuhk.edu.hk (J.Y.); siewchienng@cuhk.edu.hk (S.C.N.); 5 Diamantina Institute, Faculty of Medicine, Translational Research Institute, The University of Queensland, Brisbane 4072, Australia; m.morrison1@uq.edu.au; 6 Institute of Digestive Disease, State Key Laboratory of Digestive Diseases, LKS Institute of Health Science, the Chinese University of Hong Kong, Shatin, NT, Hong Kong SAR, China; 7 Centre for Gut Microbiota Research, The Chinese University of Hong Kong, Shatin, NT, Hong Kong SAR, China

**Keywords:** diet additive, artificial sweetener, emulsifier, Crohn’s disease, inflammatory bowel disease, survey, questionnaire, tool, assess, measure

## Abstract

(1) Background: The Food Agricultural Organization/World Health Organization (FAO/WHO) International Food Standards *Codex Alimentarius CXS 192e International Food Standards* (hereafter, CODEX) declares additives non-toxic, but they have been associated with changes to the microbiota changes and thinning of the mucus layer of the gut. Their widespread use has occurred in parallel with increased inflammatory bowel disease (IBD) incidence. This paper reports on the development and validation of surveys to estimate additive intake. (2) Methods: Dietitians created a food-additive database, with a focus on additives that have been associated with IBD. For each additive, information on the CODEX food-category they are permitted in and the associated maximum permissible levels (mg/kg) was recorded. Based on the database, questions to assess early life (part 1) and recent (part 2) additive intake were written. Forward–backward translation from English to Chinese was undertaken. Thirty-one individuals were evaluated to assess understandability. A further fifty-seven individuals completed the tool on two occasions, a fortnight apart; agreement was assessed using Cohen’s kappa coefficient or the intra-class correlation coefficient (ICC). (3) Results: The participants reported that it was difficult to remember food intake and estimate portion sizes. The participants also noted confusion around the term ‘home-grown’. Instructions and definitions were added; after this, respondents judged the questionnaires as clear. The average kappa coefficient for part 1 and part 2 questions were 0.61 and 0.67, respectively. The average ICC ranged from 0.30 to 0.94; three food lists were removed due to low reliability. (4) Conclusions: Two tools have been created and validated, in two languages, that reliably assess remote and recent food additive intake.

## 1. Introduction

Food manufacturers use additives to improve food quality or extend shelf-life. The Joint Food Agricultural Organization/World Health Organization (FAO/WHO) Expert Committee on Food Additives (JECFA) evaluates food additive safety [1]. The FAO (Food Agriculture Organization)/WHO (World Health Organization) *Codex Alimentarius CXS 192e International Food Standards* (hereafter, CODEX) details conditions for safe food additive use. CODEX assigns foodstuffs into categories (*n* = 16) and sub-categories (*n* = 138). Each additive (*n* = 301) has an International Numbering System designation (INS). For most additives, CODEX specifies a maximum permissible level (MPL) (mg/kg) for usage. The MPL ensures compliance with the acceptable daily intake (ADI). Some additives are generally regarded as safe (GRAS) and do not have an MPL. CODEX advises manufacturers to use these additives at the minimum level required, as per Good Manufacturing Practice (GMP). The aim of CODEX is to protect consumers’ health and ensure fair food-trade practices. 

The FAO/WHO food additive safety-assessments are based on toxicity [2,3]. However, emerging epidemiological evidence suggests that food additives may have biological effects that are not benign and may negatively affect the human gut microbiota and lead to destruction of the mucus layer of the gut [4]. The consumption of ultra-processed (additive-dense) and packaged (additive-containing) foods has been associated with increased all-cause mortality [5] and the increasing global incidences of obesity, metabolic syndrome, and inflammatory bowel disease (IBD) [6,7,8,9]. In Hong Kong and China, for example, IBD incidence has increased three-fold in the past decade [10], in parallel with dietary westernization [9,11] and the increased consumption of processed foods. A western diet can be typified as high in omega-3 fatty acids, saturated fat, and animal protein, and low in fibre, fruits, and vegetables [9,11]. Interestingly, in animal models of IBD, switching to a healthy diet can alleviate the detrimental effects of dietary westernization [12]. This is, in part, related to the fermentation of fiber and resultant production of anti-inflammatory short-chain fatty acids [13]. 

Recent studies suggest that the metabolic and inflammatory effects of food additives may be mediated via changes in the gut microbiota. At concentrations permitted in the food supply, emulsifiers [14,15], thickeners [16], and artificial sweeteners [17,18] change the abundance and pathogenicity of gut bacteria and perturb human [19] and animal gut mucosa [14,20,21]. 

Due to their implication in disease causation, there is growing interest in evaluating the association between dietary additives, metabolic diseases, and IBD, and exploring the therapeutic role of anti-inflammatory [22,23] and additive-free diets in IBD [24,25]. A systematic literature review of randomized controlled trials reported that no firm conclusions could be drawn regarding the efficacy of dietary interventions to induce or maintain remission in Crohn’s disease and ulcerative colitis [26]. However, the lack of a validated measure of food additive intake limits research on diet and IBD. Population-based risk studies have been undertaken [27] and researchers have used food records to estimate the average number food-additive-containing foods consumed by children with Crohn’s disease [28], but to our knowledge there are no published reports of tools to assess individuals’ food additive intake in a semi-quantitative or quantitative manner. Given the importance of the microbiota in early life [29], it is critical to be able to assess additive intake both retrospectively, currently, and prospectively. 

This paper aims to report on the use of CODEX to develop English and Chinese IBD-specific food additive surveys that can be used to assess intake of food additives in early life and over the past 12 months. A protocol for using prospective food records and food labels to estimate food additive intake is also described.

## 2. Materials and Methods 

A novel method was used to create the surveys. The steps taken are outlined in Table 1.

### 2.1. Literature Review 

The search strategy ‘IBD’ or ‘inflammatory bowel disease’ or ‘Crohn’s disease’ or ‘ulcerative colitis’ AND ‘food additive*’ or ‘diet additive*’ or ‘additive*’ or ‘nanoparticle*’ or ‘microparticle*’ was used to confirm additives that have been implicated in the development of IBD. A narrative review of factors that influence early life food intake was also conducted.

### 2.2. Development of IBD-Specific Food-Additive Database and Estimate of Annual Food-Additive Exposure

Using CODEX, two dietitians constructed an IBD-specific food-additive database. For each additive established as significant, the database included the CODEX categories which the additive can be used in, as well as the additive MPL (if available). For additives with no MPL, concentration data from published studies [15,20,30] or recommended concentrations from food technology texts [31] were used. Where conflicting concentrations were available, the highest value was taken. Food modelling was undertaken to estimate maximum additive intake from each food category (Table 2). 

### 2.3. Development of Food Additive lists

Based on the IBD food additive database, the dietitians created a master list of all CODEX food sub-categories that contained at least one additive of interest [1]. Food sub-categories were redefined using lay terminology, and where appropriate these were condensed into a single food list. Region-specific food examples for each food list were collected and cross-checked based on published literature and food labels.

### 2.4. Draft Questions to Assess Food Additive Intake 

Draft questions were written by two dietitians (G.T., W.L.) and then reviewed and edited by additional team members (M.A.K., S.C.N., W.T., J.C.). To assess food additive intake over the past 12 months (survey part 2), items were drafted to enquire about consumption of each of the food lists generated in step three. The items asked whether foods were consumed (yes/no) and if ‘yes’, how many times they were consumed (per day/week/month/year). The participants were also asked to estimate average portion or amount of food consumed. The researchers kept records of which food list contained which food additive of interest. 

A different approach was taken to assess early life food additive intake (survey part 1). Since recall could be an issue, items were kept more generic. Items were still based on the food lists developed during step three, but the lists were condensed. Additional questions to assess breastfeeding, food during infancy, and the home food environment in early life were included. To capture dietary change overtime, questions were repeated using the age-groups: four months to one year, one year to five years; >five years to 10 years; >10 years to 18 years.

### 2.5. Translations 

The English questionnaires were translated to Chinese (Cantonese-Hong Kong) and then to Chinese (Mandarin-Mainland). Translation to Chinese was undertaken based on published guidelines [32], as follows: (1) a literal (word to word) translation from English to Cantonese excluding food examples, (2) translations reconciled by expert committee, (3) a back translation to English, (4) a back translation evaluated by expert committee, (5) Cantonese translation to Mandarin occurred with cultural adaptation and refinement in vocabulary.

### 2.6. Pilot Testing

The draft surveys were pilot tested on subjects in Australian (*n* = 11), Mainland Chinese (*n*= 10), and Hong Kong (*n* = 9) samples. Subjects self-completed the survey and feedback forms. The dietitians then interviewed participants about their experience.

### 2.7. Statistical Analyses

Statistical analysis was performed using IBM SPSS Statistics for Windows, Version 24.0 (Armonk, NY, USA: IBM Corp.). New convenience samples undertook the surveys twice (one to two weeks apart). Based on an effect size 0.8, kappa 0.85 (strong agreement), alpha level 0.05, the required sample size was calculated as 44 persons on each attempt [33]. This was in line with Consensus-based Standards for the selection of health Measurement Instruments (COSMIN) recommendations that a sample of 50 is regarded as ‘good’ for test re-test assessment [34]. 

Part 1: Reproducibility of individual questionnaire items was assessed using Cohen’s kappa coefficient. Cohen’s kappa evaluates the beyond-chance agreement between two ratings [32]. Values are interpreted as follows: 0.01–0.20 none to slight; 0.21–0.40 fair; 0.41–0.60 moderate; 0.61–0.80 substantial; and 0.81–1.00 almost perfect [35]. For each question, the average percent of respondents who answered in an identical manner on each attempt was also calculated. Likewise, for each person, the total percent of questions answered in an identical manner between attempts was calculated. Having more than 75% of questions answered in a consistent manner was taken to indicate adequate reliability.

Part 2: For continuous variables (times per year each ‘food list’ on the part 2 survey was consumed, agreement was assessed using the intra-class correlation coefficient (ICC)). A two-way mixed effects model for single measurement agreement was used. Values less than 0.5, between 0.5 and 0.75, between 0.75 and 0.9, and greater than 0.90 are indicative of poor, moderate, good, and excellent reliability, respectively [36]. Existing Food Frequency Questionnaires (FFQs) have a reliability coefficient between 0.2 and 0.9 [36,37,38,39]. 

### 2.8. Ethics Approval and Consent to Participate

All described procedures were approved by St Vincent’s Hospital, Melbourne, Australia, Quality and Risk Unit (QA 025/18). For the pilot study, participants provided verbal consent due to the below low-risk nature of the project and as deemed appropriate by St Vincent’s Hospital Quality & Risk Unit. For the statistical validation, informed consent was provided via continued completion of online questionnaires, as deemed appropriate by St Vincent’s Hospital Quality and Risk Unit.

## 3. Results

### 3.1. Litreature Review

Additives of interest: At the time of conducting the literature search, ten additives had been implicated as potential causative agents in IBD. These include the thickener Maltodextrin [16,40] (not covered by CODEX), three emulsifiers and/or thickeners (carrageenan [41], carboxymethylcellulouse [11], polysorbate-80 [15]), three artificial sweeteners (aspartame [42], saccharin [43], sucralose [44]), two nanoparticles (the coloring agent titanium dioxide [30,45] and the anti-caking agent sodium aluminosilicate [45,46]), and sulfite preservatives [47]. Factors influencing early life food intake: Factors that impact exposure to food additives in childhood were extrapolated from a model of the home food environment pertaining to childhood obesity [48]. Key influencers that were found to be relevant to additive intake were access and availability of fresh food, kitchen equipment, and the presence of a home garden.

### 3.2. Construct IBD-Specific Food-Additive-Database 

An IBD database was constructed. Additive concentrations ranged from 20 mg/kg (sulphites in pasta) to 10,000 mg/kg (aspartame in chewing gum). 

### 3.3. Estimation Additive Exposure and Develop Food Additive Lists

The database was used to estimate and food additive exposure and develop food additive lists. An example of food modelling to estimate the maximum additive exposure is shown in Table 3.

### 3.4. Draft Questions to Assess Food Additive Intake

The first draft of the early life food additive survey (part 1) had 39 dichotomous questions on foods eaten up to the age of 18 years. There are three items related to breastfeeding, the intake of processed baby food, and the place in which the food was purchased. Nine questions, repeated for each age group older than 4 months, covered the availability of home-grown food (*n* = 1) and the intake of processed foods (*n* = 8). Items on processed foods covered intake of fast food, soft drinks, and savory snacks, as well as packaged foods from each of the five core food groups (dairy, meat, grains, fruits, and vegetables). The first draft of the ‘current’ food additive survey (part 2) had 24 ‘food lists’. The questions asked if the food list was consumed (yes/no). If yes, the frequency (per day, week, month, and year) and amount consumed were included.

### 3.5. Translations

Three versions were generated: (1) an English, (2) Chinese (Hong Kong Traditional Chinese), and (3) Chinese (Mainland Simplified Chinese) version. There was good agreement during the forward–backward translation process. 

### 3.6. Pilot Testing 

Thirty-one individuals undertook the survey as part of a pilot study to assess understandability. The completion rates were 100%. Several participants had issues recalling early life food intake. They asked whether ‘home-grown’ fruits and vegetables included fresh options purchased at the store and if supermarket bread, tea, and coffee were store-bought. They also suggested common examples of foods as additions to food lists. Participants reported trouble recording quantities of foods eaten for the part-two survey. 

Based on this feedback, several modifications were made. For the part 1 survey, the instructions were expanded to include: “If you think you had the following foods or drinks EVERY WEEK in the following age groups, tick ‘Yes’ for questions 5 to 12. We understand this may be difficult to recall, but please make the best guess possible.” In addition, the statement “Home-grown fruits and vegetables are those grown by yourself” was added to the question on consumption of home-grown fruits and vegetables.

For the part 2 survey, instructions were added to explain how to estimate the average amount of food consumed, as follows: “Please estimate using household measures e.g., 1 Tablespoon, 1 cup or other common description of food sizes e.g., 1 palm size, 1 fist full”.

In addition, store-bought was added to the description of bread, egg-based dessert; bottled tea/bottled coffee and coffee substitutes was added to the description of tea and coffee; ‘tea and coffee’ and ‘bread and crackers’ were separated into two food lists (as individuals reported different consumption patterns for these) and several examples were added to food lists.

### 3.7. Statistical Validation

One hundred and eight participants were invited to complete the surveys. The test-retest completion rates for part 1 and part 2 were 54% and 44%, respectively. The average time between completion of surveys was 12 days. One person was excluded from the part 2 analysis as their responses on the second attempt were judged to be implausible. The sample was 61% Australian and 77% female. The mean age of respondents was 37 years (range 23–74 years).

Part 1: The percent agreement for the 39 items ranged from 72% to 100% (mean 86%). All but one item had an acceptable percent agreement between responses. For two items, the kappa coefficient could not be calculated due to high similarity between response patterns. The kappa coefficient for the remaining items ranged from 0.29 to 0.96 (mean 0.61), with all values being statistically significant. Three items had fair kappa coefficient, 21 items had moderate kappa coefficient, 12 items had substantial kappa coefficient, and the item on breast-feeding had an almost perfect kappa coefficient.

Items on place of shopping and fast food consumption in the first year of life also had very strong correlations. Items on food at infancy (home-made versus store-bought), the consumption of processed dairy after the first year of life, and the consumption of processed fruit in the first year of life had weak correlations (Table 4). 

For individuals, the mean percent agreement between attempts ranged from 59% to 100%. Ninety-three percent of respondents had a mean percent agreement above 75%. The removal of the respondents with low percent agreement between attempts did not significantly change the results. No changes were made to the part 1 questionnaire as a result of the validation. 

Part 2: The ICC for the total, combined annual frequency of the 26 food lists was 0.999 (*p* < 0.001). The ICC for individual food lists ranged from 0.30 to 0.94, the average ICC was 0.61. Twelve lists had moderate reliability, five had good reliability, and three had excellent reliability. Six lists had poor reliability, however, of these, processed meat (0.48), egg-based desserts (0.48), and coffee (0.45) had values approaching the requisite value for a moderate correlation. The remaining food lists with poor reliability were those assessing the intake of sugars and syrups (0.12), store-bought pasta (0.30), and flavored milk (0.41) (Table 5).

The list on flavored milk was retained, as the relatively low ICC value could be explained by a couple of participants who drastically changed their responses between survey attempts. For example, one participant said they had ‘no’ flavored milk on attempt one and that they consumed this ‘three times per day’ on attempt two and the other said none versus one daily. With these anomalies removed, the values for flavored milk were 0.78 (*p* < 0.001). Furthermore, one individual said they drank ‘no’ coffee and ‘4 cups a day’ on two occasions; when this response was removed from analysis the value for coffee increased from 0.48 to 0.81 (*p* < 0.001). The pasta, sugar, and syrups list(s) were removed from the final tool. In addition to a poor ICC, these foods rarely contained additives of interest. 

### 3.8. Scoring System 

Part 1: The part one survey has dichotomous responses and is therefore not detailed enough to estimate additive intake in a quantitative manner. Rather than using the questions to estimate intake (in mg), the items are designed to be scored as 0 (exposure less likely) or 1 (exposure more likely). The total scores can be used to rank participants by early-life food additive intake. 

Part 2: The part 2 survey can be used in a semi-quantitative manner to rank participants in terms of general food additive exposure based on ‘frequency per year’ for each food additive list. Sub-scores can be calculated for additive classes based on which additives are permitted in which food lists. Although manufacturers do use multiple additives in the same class (e.g., three to four different emulsifiers) in a single food product, often a lower concentration will be used when another additive with a similar purpose is present. Therefore, to avoid overestimation when calculating a semi-quantitative additive sub-score, the sub-scores should be based on the class of additive rather than individual additives. Maximal exposure to food additives can also be estimated in mg/year using methods akin to those used to construct the food additive database. That is, where a food additive is permitted in a given list, the MPL (or concentration data from the literature [15,20,30,31]) can be multiplied by estimated amount of food (in g) consumed per year to estimate the annual maximum exposure to the additive. An example is demonstrated below using the ‘Sports Drinks’ food list (Table 6). Caution needs to be taken when using this approach, however, as permitted additives are not always present in foods, and if present may be used in amount below the MPL.

### 3.9. Using the Database to Estimate Food Additive Intake from Food Diaries

Where food diaries are available, CODEX and the food additive database can be used to estimate food additive intake. Food diaries need to be detailed and include the brand names of all foods. Once the average amount per day (g) of each packaged food is calculated, information on the presence or absence of additives of interest (based on ingredients list) needs to be documented. Foods that contain at least one additive of interested need to be classified into a relevant CODEX food category. The amount of the additive in the food can then be calculated using the IBD food additive database. An example can be seen below (Table 7). 

## 4. Discussion

This study reports the development and validation of two surveys to estimate food additive intake. The final part 1 survey has 12 questions (nine with four parts, for a total of 39 items) on breastfeeding, food during infancy, and the consumption of processed foods and drinks in early life. The final part 2 survey is a food-frequency survey; the Australian version has 24 food lists, the Hong Kong version has 25 food lists, and the Mainland version has 25 food lists. Methods for estimating food additive exposure using food labels have also been described. 

Multiple surveys exist for estimating nutrient and food intake, but these are the first surveys designed to estimate an individual’s food additive intake in a semi-quantitative manner. We chose the foods in the survey based on a systematic approach that considered allowed levels of additives relevant to IBD. The surveys are appropriate for culturally diverse populations, making them well suited to epidemiological studies [49]. The tools can be self-administered but should be checked by researchers. For example, it should be clear that ‘flavored milk’ does not include plain cow’s milk. Researchers should ensure participants have filled the correct line and boxes, and can use household measures, measuring cups, images, and food models to assist with the estimation of portion sizes [50].

Validation for most included items had moderate to strong correlations. The kappa coefficient for part 1 ranged from 0.30–0.96 (percent agreement of 72% to 98%) and the ICC for the included lists in part 2 ranged from 0.45 to 0.92. In a food frequency questionnaire (FFQ) designed to assess the usual food consumption of women in South East China, the ICC for food groups ranged from 0.44 to 0.96 and the kappa coefficient for eating habits ranged from 0.27 to 0.89 [37]. An FFQ designed for women in Guangdong reported adjusted Pearson correlations of 0.30 to 0.68 for food groups [51]. The Dietary Questionnaire for Epidemiological Studies (DQESV2) [38], a common FFQ used in Australia, reported ICC for fruits and vegetables ranging from 0.54 to 0.86. The Australian Eating Survey FFQ was assessed for reproducibility, however, values for estimated nutrients (not actual food lists) were reported. The kappa statistics ranged from 0.57 to 0.81 [36]. A literature review by [39] found that FFQs generally have reliability coefficients of 0.5–0.7. Thus, the reproducibility of the tools developed in this study appears to be in line with existing dietary assessment tools. 

There are several limitations associated with measuring dietary intake, which include a recall bias and a social desirability bias [52]. Using prospective methods to collect dietary data (such as food diaries) partly overcomes issues associated with the recall bias but is associated with a greater respondent burden and a chance that participants will alter their behavior [53]. The recall bias may increase in studies looking at intake further into the past, such as for our part 1 survey. Several studies have assessed individuals’ (or their parents’) ability to estimate intake from 10 to 40 years earlier. The correlation coefficients between recalled and prospectively recorded intake range from 0.07 to 0.81, and average around 0.5 [54,55], indicating a moderate, positive association. Similar associations occur when assessing food frequency questionnaire (FFQ) against weighed food records (the gold standard assessment method for food intake [39]). Techniques to assist recall should be employed when using retrospective measures of food intake [56]. 

In addition to the general limitations associated with assessing food intake, the measurement of food additives is very complex. There are thousands of food additives in use and a growing and changing food supply. Food additive use by manufacturers varies greatly even across similar foods and the concentration of additives in most foods is not reported. Moreover, due to labeling laws, additives used during manufacture are not always declared. We based some estimations on the MPL set by the FAO/WHO. This approach will overestimate intake because manufacturers may use permitted additives at levels below the MPL (or not at all). Some of the food lists included in our tools, such as ‘milky desserts’, ‘sweets and lollies’, and ‘sweet baked foods’ are very broad, and there is likely to be a large variety of additives used in these foods. Likewise, the results from the coffee and crackers list need to be interpreted with caution because foods in these categories often do not contain any additives. Canned foods (fish/fruit/vegetables) also often do not contain any additives but may contribute to aluminum or microplastic intake. 

Several foods not included, namely take-away, gluten-free foods, low-fat foods, and vegetarian alternatives and mock-meat products (such as those based on seitan and mycoprotein) frequently contain food additives. These will be added to future versions of the survey. Additionally, possible pesticide exposure [57] will be added. A question asking “How often do you choose organic fruits and vegetables”, with the options “Always/Mostly/Sometimes/Never” will be added. Details about trehalose intake will also be added. Trehalose is a disaccharide added to chewing gum and processed-diet foods that has been shown to increase virulence of Clostridiodes difficile [58]. 

Future studies should also focus on assessing construct validity by comparing estimates from the FFQ and three- to five- day food records. The inclusion of pharmaceuticals and cosmetics that contain additives may also be relevant. Finally, the questionnaires could be validated against objective markers, such as the presence of additive metabolites in biological samples. 

## 5. Conclusions 

This study reports on the development and validation of two surveys that assess the remote and current intake of food additives. The part 1 survey assesses remote food additive exposure based on whether subjects were breastfed, had fresh food available to them, or frequently consumed additive-containing foods. The part 2 survey assesses current food additive intake based on frequency and quantity of additive-containing foods. The surveys have been judged as easy to understand and complete based on a pilot validation study. They have acceptable reliability. Methods for scoring the surveys to estimate food additive intake have been described. Content validation will be undertaken in future studies. These tools can be employed in the important field of research evaluating the role of food additives in the development and disease course of IBD. 

## Figures and Tables

**Table 1:** Steps taken to design and evaluate food additive surveys.

Step one: Literature review Step two: Develop IBD-specific food-additive-database and estimate annual food additive exposure Step three: Develop food additive lists Step four: Draft questions to assess food additive intake Step five: Translations Step six: Pilot testing Step seven: Statistical validation

**Table 2:** Formula used to estimate exposure to food additives.

Annual exposure = additive food concentration (mg/kg) ^a^/1000 × food amount per day (g) ^b^ × 365 = mg/year (a) Based on maximum permissible level allowed in food (CODEX) OR actual amount in food (based on literature review of analytical studies) (b) Based on the following assumptions: (i) 1 standard serve/day for ‘non-core’ foods or (ii) recommended serves/day for ‘core’ food (fruit, vegetable, minimally processed grain e.g., rice, fresh meat) then calculated based on recommended serves/day

**Table 3:** Example of Food Additive Modelling Using Aluminosilicate.

Codex Category Number	Codex Food Category Description	Additive MPL (mg/kg)	Serve Size (g)	Estimated Additive (mg)	Serves/day	Estimated Yearly Exposure (mg)
01.8.2	Dried whey and whey products	1140	30	34.2	1	12483
12.5.2	Mixes for soups and broths	570	50	28.5	1	10402.5
12.6.3	Mixes for sauces and gravies	570	50	28.5	1	10402.5
01.5.2	Milk and cream powder analogues	570	30	17.1	1	6241.5
12.1.1	Salt	1000	2.3	2.3	1	839.5
5.3	Chewing gum	100	2.8	0.28	1	102.2
01.3.2	Beverage whiteners—lowest level in analysed foods	3	12	0.036	1	13.14
12.2.2	Seasonings and condiments	1000	5	0.005	1	0

**Table 4:** Percent agreement and Kappa Coefficient for items on part on survey.

Question	Percent Agreement	Kappa Coefficient	p^2^
Breast fed	98.3	0.959	<0.001
Food at infancy	77.6	0.311	<0.05
*Home grown produce*			
3a. 4 month-1 year	86.2	0.566	<0.001
3b. 1–5 years	91.4	0.536	<0.001
3c. 5–10 years	89.7	0.659	<0.001
3d. 10–18 years.	89.7	0.760	<0.001
Place of shopping	98.3	0.701	<0.001
*Processed dairy consumption*			
5a. 4 month-1 year	89.7	0.703	<0.001
5b. 1–5 years	75.9	0.421	<0.001
5c. 5–10 years	72.4	0.377	<0.05
5d. 10–18 years.	77.6	0.470	<0.001
*Processed meat consumption*			
*6a. 4 month-1 year*	93.1	Could not calculate	
6b. 1–5 years	81.0	0.504	<0.001
6c. 5–10 years	77.6	0.502	<0.001
6d. 10–18 years.	81.0	0.611	<0.001
*Processed grain consumption*			
7a. 4 month-1 year	84.5	0.439	<0.001
7b. 1–5 years	93.1	0.661	<0.001
7c. 5–10 years	91.4	0.685	<0.001
7d. 10–18 years.	93.6	0.570	<0.001
Processed fruits consumption			
8a. 4 month-1 year	84.5	0.298	<0.001
8b. 1–5 years	93.1	0.552	<0.001
8c. 5–10 years	98.3	0.746	<0.001
8d. 10–18 years.	84.5	Could not calculate	
*Processed vegetables consumption*			
9a. 4 month-1 year	84.5	0.499	<0.001
9b. 1–5 years	82.8	0.552	<0.001
9c. 5–10 years	77.6	0.520	<0.001
9d. 10–18 years.	81.0	0.595	<0.001
*Fast food consumption*			
10a. 4 month-1 year	100	Could not calculate	
10b. 1–5 years	84.5	0.512	<0.001
10c. 5–10 years	84.5	0.679	<0.001
10d. 10–18 years.	87.9	0.757	<0.001
*Soft drinks consumption*			
11a. 4 month-1 year	94.8	0.374	<0.001
11b. 1–5 years	89.7	0.731	<0.001
11c. 5–10 years	86.2	0.724	<0.001
11d. 10–18 years.	87.9	0.736	<0.001
*Snacks consumption*			
12a. 4 month-1 year	81.0	0.580	<0.001
12b. 1–5 years	77.6	0.533	<0.001
12c. 5–10 years	81.0	0.417	<0.001
12d. 10–18 years.	86.2	0.583	<0.001

**Table 5:** Intra Class Correlation coefficients (ICC) for food lists in part 2 food additive survey.

Food List	ICC	p^2^
1. Store-bought pasta, pasta salad, or noodles (e.g., packet spaghetti, pre-made ravioli, pre-made pasta salad)	0.30	<0.001
2. Supermarket bread (e.g., packaged sliced bread, packaged wraps)	0.50	<0.001
3.. Crackers (e.g., rice-cakes, savory crackers)	0.73	<0.001
4.Packaged soup (e.g., Cup-of Soup, broth in a can, soup mixes by Heinz or Campbell)	0.97	<0.001
5. Processed meats and products (e.g., deli meat, sausages, burgers, chicken nuggets)	0.48	<0.001
6. Processed seafood and products (e.g., canned clams, canned salmon, fish fingers, fried seafood, crab cakes)	0.77	<0.001
7. Processed vegetables (e.g., canned vegetables, pickled vegetables, fermented vegetables, vegetable juice	0.64	<0.001
8. Processed fruits and products (e.g., dried fruit, canned fruit, fruit compote, jam, fruit juice)	0.69	<0.001
9. Flavored milk (e.g., Moo™ chocolate milk, Dare™ iced coffee)	0.41	<0.001
10. Processed cream products (e.g., sour cream, sour milk, kefir, pouring cream, whipped cream)	0.67	<0.001
11. Milky desserts (e.g., chocolate mousse, vanilla pudding, flavored yoghurt)	0.70	<0.001
12. Sugars and syrups (e.g., golden syrup, maple syrup, sugar toppings)	0.12	<0.05
13. Chewing gum (e.g., Extra™)	0.69	<0.001
14. Sweet baked foods (e.g., cakes, biscuits, muesli bars, gluten-free cake)	0.75	<0.001
15. Bottled Tea	0.58	<0.001
16. Coffee and coffee substitutes (e.g., instant coffee, espresso coffee, Echo™)	0.45	<0.001
17. Sports Drinks (e.g., Powerade™, Gatorade™)	0.54	<0.001
18. Diet drinks or sugar substitutes (e.g., Diet Coke™, Pepsi-max™, diet iced-tea, drinks sweetened with Equal™, Splenda™, Sweet ‘n’ Low™)	0.82	<0.001
19. Alcoholic drinks (e.g., beer, wine, cider, spirits)	0.94	<0.001
20. Vitamin pills (e.g., fish oil capsules, multivitamin pills)	0.59	<0.001
21. Whey proteins and products (e.g., protein bar, protein powder)	0.52	<0.001
22. Egg-based desserts (e.g., custard)	0.48	<0.001
23. Milk powder (e.g., instant dry milk)	0.67	<0.001
24. Salad dressing (e.g., mayonnaise, tartar sauce, Thousand Island dressing)	0.77	<0.05
25. Sweets and lollies (e.g., liquorice, mints, skittles, marshmallows, chocolates)	0.83	<0.001
26. Coffee-whitener (e.g., Coffee-mate™)	0.92	<0.001

**Table 6:** Example of using part 2 questionnaire to estimate maximum food additives consumed.

Food List	CODEX Category (number)	Participants Intake	IBD Additives (mg/kg)	Estimated Intake (mg)
Sport drink	Sport drinks (14.1.4)	1200 mL per month	Sulfites (70) Aspartame (400) Sucralose (600)	1008 5760 8640

You could also use responses to the part 2 questions to calculate a semi-quantitative score based on times/year sports drinks were consumed, giving a score of ‘24′. Likewise, sub-scores could be calculated by attributing ‘points’ for each additive which is permitted in the food list; for example, for sports drinks, sub-score points would be added to the preservative and artificial sweetener sub-groups.

**Table 7:** An example of using CODEX to estimate maximum additive consumed in a food.

Food (g)	CODEX Category (number)	IBD-Food Additive (E number)	Additive MPL (mg/kg)	Consumed (mg)
Praise deli-style dijonaise (20)	Emulsified sauces and dips (12.6.1)	(1) Sulphites (E223)	350	7
		(2) Titanium dioxide (E171) (3) Carboxymethylcellulouse (E466)	7500 No MPL; used at concentrations of 0.1–0.5%	150 10 (based on 0.5% *w*/*v*)
